# Hydrogen self-diffusion in single crystal olivine and electrical conductivity of the Earth’s mantle

**DOI:** 10.1038/s41598-017-05113-6

**Published:** 2017-07-13

**Authors:** Davide Novella, Benjamin Jacobsen, Peter K. Weber, James A. Tyburczy, Frederick J. Ryerson, Wyatt L. Du Frane

**Affiliations:** 1 Lawrence Livermore National Laboratory, Livermore, California 94550 USA; 2 Department of Earth Sciences, University of Cambridge, Downing Street, Cambridge, CB2 3EQ UK; 3 School of Earth and Space Exploration, Arizona State University, Tempe, Arizona 85287 USA

## Abstract

Nominally anhydrous minerals formed deep in the mantle and transported to the Earth’s surface contain tens to hundreds of ppm wt H_2_O, providing evidence for the presence of dissolved water in the Earth’s interior. Even at these low concentrations, H_2_O greatly affects the physico-chemical properties of mantle materials, governing planetary dynamics and evolution. The diffusion of hydrogen (H) controls the transport of H_2_O in the Earth’s upper mantle, but is not fully understood for olivine ((Mg, Fe)_2_SiO_4_) the most abundant mineral in this region. Here we present new hydrogen self-diffusion coefficients in natural olivine single crystals that were determined at upper mantle conditions (2 GPa and 750–900 °C). Hydrogen self-diffusion is highly anisotropic, with values at 900 °C of 10^−10.9^, 10^−12.8^ and 10^−11.9^ m^2^/s along [100], [010] and [001] directions, respectively. Combined with the Nernst-Einstein relation, these diffusion results constrain the contribution of H to the electrical conductivity of olivine to be σ_H_ = 10^2.12^S/m·C_H2O_·exp^−187kJ/mol/(RT)^. Comparisons between the model presented in this study and magnetotelluric measurements suggest that plausible H_2_O concentrations in the upper mantle (≤250 ppm wt) can account for high electrical conductivity values (10^−2^–10^−1^ S/m) observed in the asthenosphere.

## Introduction

Earth’s hydrosphere is a distinctive feature of our planet where massive oceans affect its climate and support its ecosystem. The distribution of H_2_O on Earth is not limited to its outermost shell (hydrosphere and hydrated minerals), but extends to great depths within the planet. Downwelling oceanic lithosphere (at subduction zones), and upwelling magmas (at mid ocean ridges, volcanoes and hotspots) are vehicles for transport of H_2_O between the surface and the Earth’s deep interior^1^. Experimental studies have shown that substantial concentrations of H_2_O may be present in the mantle, hosted as defects in the structure of nominally anhydrous minerals (NAMs). The major mineral component of the uppermost mantle, olivine ((Mg, Fe)_2_SiO_4_), can incorporate as much as ~1 wt% H_2_O at conditions encountered at the lowest portion of the upper mantle^2–4^. Minerals found in mantle xenoliths, including olivine, that are brought to surface by alkaline or kimberlitic magma eruptions, contain up to hundreds of ppm wt (parts per million by weight) H_2_O^5^, providing direct evidence of H_2_O incorporated in NAMs from the Earth’s interior. Recently, a crystal of terrestrial ringwoodite (a high-pressure polymorph of olivine that forms below ~410 km depth), discovered as an inclusion in a natural diamond, was found to contain ~1.4 wt% H_2_O^6^. This confirms that at least some regions within Earth’s transition zone contain large concentrations of dissolved H_2_O.

H_2_O, as hydrogen (H) bonded to the crystal structure, can influence the physical and chemical properties of minerals even if only present at ppm wt levels^7^. For example, H influences the rheological properties of minerals and has been proposed as a possible factor in weakening H-bearing olivine in the convective mantle while relatively dry olivine is thought to stabilize the mantle beneath continents^8^. Furthermore, H enhances the formation of fluids/melts within the Earth by lowering the melting temperature of mantle minerals^9^, affecting the chemical differentiation of the planet. Therefore, constraining the presence and transport of H in the Earth’s mantle is crucial to understanding planetary evolution and geodynamics.

Knowledge of the electrical conductivity of mantle minerals is critical for interpreting magnetotelluric sounding measurements used to interrogate the structure and composition of the Earth’s interior. The diffusion of H in olivine is very fast^10–12^, relative to other cations and oxygen, resulting in a substantial contribution to its electrical conductivity^13^. Due to the high sensitivity to H content, electrical conductivity can thus be envisaged as a tool to investigate the H_2_O contents throughout the mantle.

The influence of H_2_O on the electrical conductivity of olivine has been measured in high pressure (P) and high temperature (T) experiments. Two recent studies agreed that H enhances olivine’s electrical conductivity, but reached contrasting conclusions regarding the implications of this enhancement with respect to measurements of mantle electrical conductivity^14, 15^. High electrical conductivity anomalies (10^−2^–10^−1^ S/m) are observed in the asthenosphere^16–18^, the region directly beneath the lithosphere where the upper mantle becomes relatively ductile. Yoshino *et al*.^14^ concluded that hydrous olivine is not capable of explaining these high electrical conductivity values, while Wang *et al*.^15^ concluded that limited amounts of H (~80 ppm wt H_2_O) dissolved in olivine are sufficient to match these geophysical measurements. The use of different analytical techniques and calibrations to determine sample H_2_O contents complicates direct comparisons of the measurements from various groups^19^. Moreover, the use of different experimental procedures may have affected the electrical conductivity measurements and also could be to blame for the apparent disagreements between datasets^20–22^. Despite substantial follow-on work devoted to determining the influence of H on olivine and mantle conductivity^23–26^, the disparate interpretations remain unresolved.

Hydrogen self-diffusion in olivine provides an alternative method for determining electrical conductivity that circumvents some of the experimental difficulties associated with previous *in-situ* electrical conductivity measurements. The Nernst-Einstein relation can be used to relate generalized mobility of H to its electrical mobility^13^. In the absence of a melt or fluid phase, the diffusion of H in mantle minerals, and along their grain boundaries, controls its distribution in the Earth’s interior. Chemical diffusion of H in olivine, i.e. the diffusion due to a concentration gradient, has been investigated by a number of groups^10–12, 27^. Hydrogen self-diffusion (i.e. the intrinsic mobility of protons in the absence of a chemical gradient) rate-limits chemical diffusion and is poorly constrained, limiting our knowledge of hydrogen mobility in the Earth’s mantle. Two studies have investigated H self-diffusion coefficients in enstatite^28^ and ringwoodite^29^. One recent study reports H self-diffusion coefficients in natural olivine along the fast [100] direction, but was unable to resolve profiles in the slow [010] and [001] directions^30^. The lack of experimental data is mainly due to (i) the challenges in successfully performing consecutive, multi-step self-diffusion experiments at elevated P-T conditions^28–30^ and (ii) the spatial resolution required to analyze diffusion profiles along both the fast and slow diffusion directions. Hydrogen diffusion profiles can be hundreds or tens of microns in length within the same sample of highly anisotropic olivine^30^.

Here, for the first time, H self-diffusion coefficients (D_H_) in single crystal olivine are reported for all three principal crystallographic orientations. Results were obtained at 2 GPa and between 750–900 °C via H-D (hydrogen-deuterium or ^1^H-^2^H) exchange experiments followed by high spatial resolution analysis of the resulting isotopic profiles utilizing Secondary Ion Mass Spectrometry (NanoSIMS). The new results better constrain the effect of H on olivine electrical conductivity for all orientations, and reconcile some of the discrepancies in previously reported results. A diffusion-based model of electrical conductivity is used to infer that olivine H_2_O contents comparable to those expected for mid-ocean ridge basalt (MORB), and below ocean island basalt (OIB), mantle sources can explain electrical conductivity anomalies observed in the asthenosphere^16–18^.

## Results

Diffusion profiles for ^2^H (D) were successfully measured along [100], [010] and [001] directions of samples cut from a single crystal of olivine. The samples were prepared using a three-step procedure consisting of a ‘dry’ anneal at high T and controlled *f*O_2_, a ‘wet’ anneal to saturate the samples with hydrogen at high P and T, and a final ‘exchange’ anneal for the isotopic exchange at high P and T (see Methods and Du Frane and Tyburczy)^30^.

Two mechanisms have been proposed for incorporation of hydrogen into Fe-bearing olivine: (1) redox exchange with positively charged small polarons (i.e. electron holes associated with Fe^3+^) accommodated by existing metal vacancy sites, or (2) slower concurrent diffusion of negatively charged metal vacancies into the crystal (i.e. creation of new sites)^11^. The ‘dry’ anneal step, >16 hrs at 1300 °C and *f*O_2_ close to that of a Ni-NiO buffer (following the experimental procedure in Du Frane and Tyburczy^30^) is used to establish a homogeneous, equilibrium concentration of small polarons and metal vacancies throughout the crystal lattice^11^. During the wet anneal at 2 GPa and 750–900 °C, hydrogen is then expected to enter the crystal via the more rapid mechanism of redox exchange with polarons on metal vacancy sites that were established during the prior dry anneal step^11^. Then in the final step, deuterium is exchanged with hydrogen established in the wet anneal. The center of the single-crystal sample PC28 (Table 1) was determined to contain 74 ppm wt H_2_O after deuterium-hydrogen exchange^31^, which is similar to the H_2_O contents of olivine samples saturated at similar conditions (2 GPa, 950 °C, for 48 hours) using the same capsule assembly^32^. H_2_O concentrations of samples PC25 and PC33 are expected to be similar because their dry anneal steps were performed at the exact same conditions, and their wet anneal steps were performed at only slightly higher or lower temperatures.

**Table 1:** H self-diffusion coefficients in olivine at 2 GPa.

Run #	T (°C)	time (h)	Log D_H,[100]_^c^	Log D_H,[010]_^c^	Log D_H,[001]_^c^
PC25	750^a^	17/48/1^b^	−12.41 (0.05)^d^	−13.85 (0.10)	−13.10 (0.05)
PC28	800	16.5/26/0.25	−11.71 (0.15)	−13.22 (0.05)	−12.75 (0.05)
PC33	900	17/18/0.17	−10.94 (0.15)	−12.75 (0.15)	−11.90 (0.05)

^a^Temperature of the wet and exchange experiments^30^; ^b^durations of dry/wet/exchange experiments^30^; ^c^D in m^2^/s; ^d^value in parentheses is 1 standard deviation (see Figure S3).

**Figure 1:**
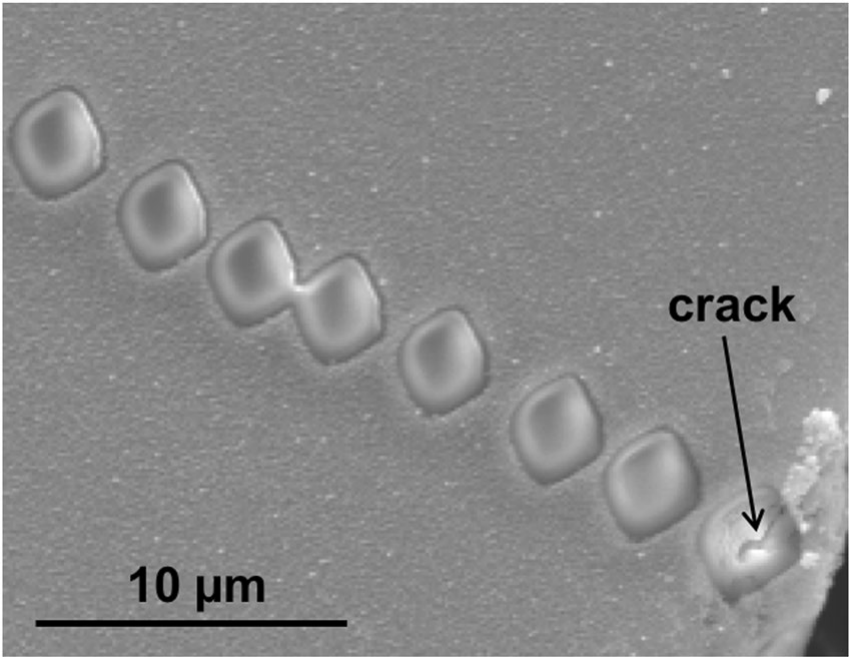
Back-scattered electron image showing NanoSIMS ion beam craters for the [001] profile on olivine PC25 (see Table 1). A crack passing through the first NanoSIMS crater from the crystal edge (bottom-right) was recognized and this analysis was discarded.

**Figure 2:**
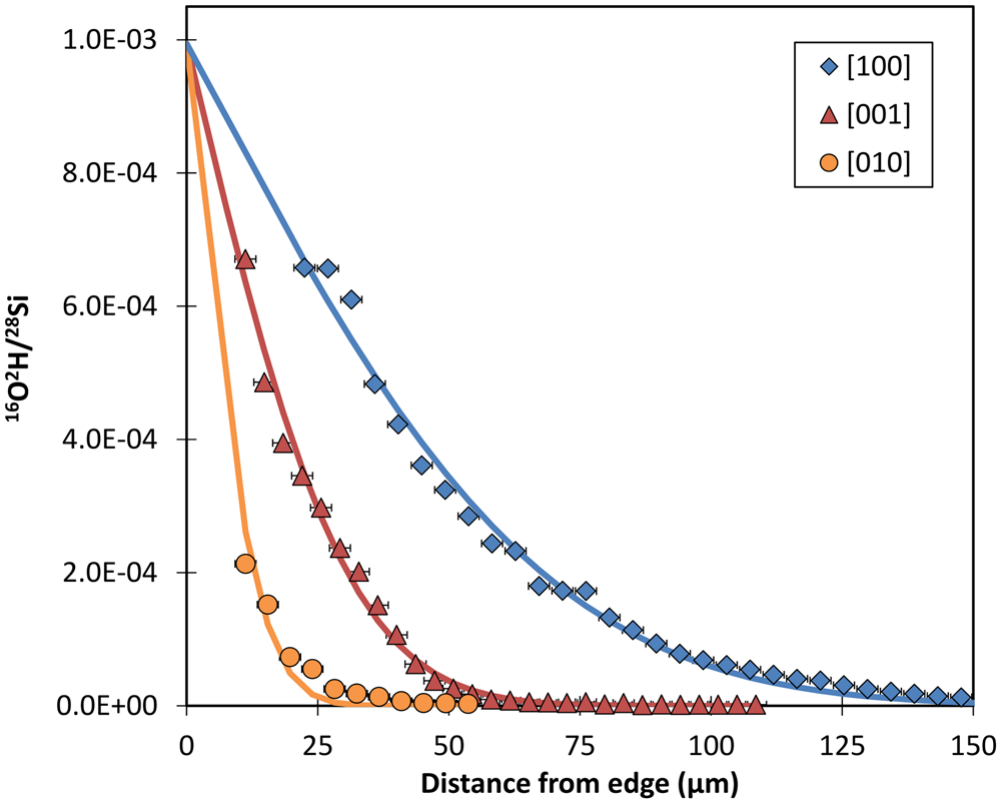
Deuterium diffusion profiles (plotted as ^16^O^2^H^−^ /^28^Si^−^, which is directly proportional to deuterium concentration in olivine) as a function of distance normal to faces oriented along [100] (blue diamonds), [001] (red triangles) and [010] (yellow circles), with lines showing fits (equation 1, see text). Data are for experiment PC25 (2 GPa, 750 °C). Average background ^16^O^2^H^−^ /^28^Si^−^ values were monitored to be 10^−5^–10^−6^ by measuring nominally anhydrous San Carlos olivine, considered to be a blank for ^2^H. Diffusion profiles along all principal orientations were observed to decrease to background noise values at approximately 150, 60 and 30 µm for this experiment, depending on orientation.

The ^16^O^2^H^−^ /^28^Si^−^ profiles, which are directly proportional to deuterium concentration in olivine, were fit to the solution to Fick’s second law of diffusion for a semi-infinite solid with a constant surface concentration^33^ to obtain H self-diffusion coefficients (Table 1). This relation describes the variation of concentration (C) of a species as a function of position perpendicular to a surface of the crystal (x) and time (t),

1 $$({{\rm{C}}}_{({\rm{x}},{\rm{t}})}-{{\rm{C}}}_{0})/({{\rm{C}}}_{1}-{{\rm{C}}}_{0})=\text{erfc}(x/(2\surd ({{\rm{D}}}_{{\rm{H}}}{\rm{t}}))$$

where C_0_ is the initial concentration of the species, set to zero, and C_1_ is the concentration at the edge of the sample. The distance of each NanoSIMS crater from the crystal edge (Fig. 1) was accurately determined using high-resolution scanning electron microscopy (SEM). The diffusion profiles for the three principal orientations of each sample were fit by regression using the same value of C_1_ (Fig. 2 and Supplementary Figures S1, S2 and S3).

**Figure 3:**
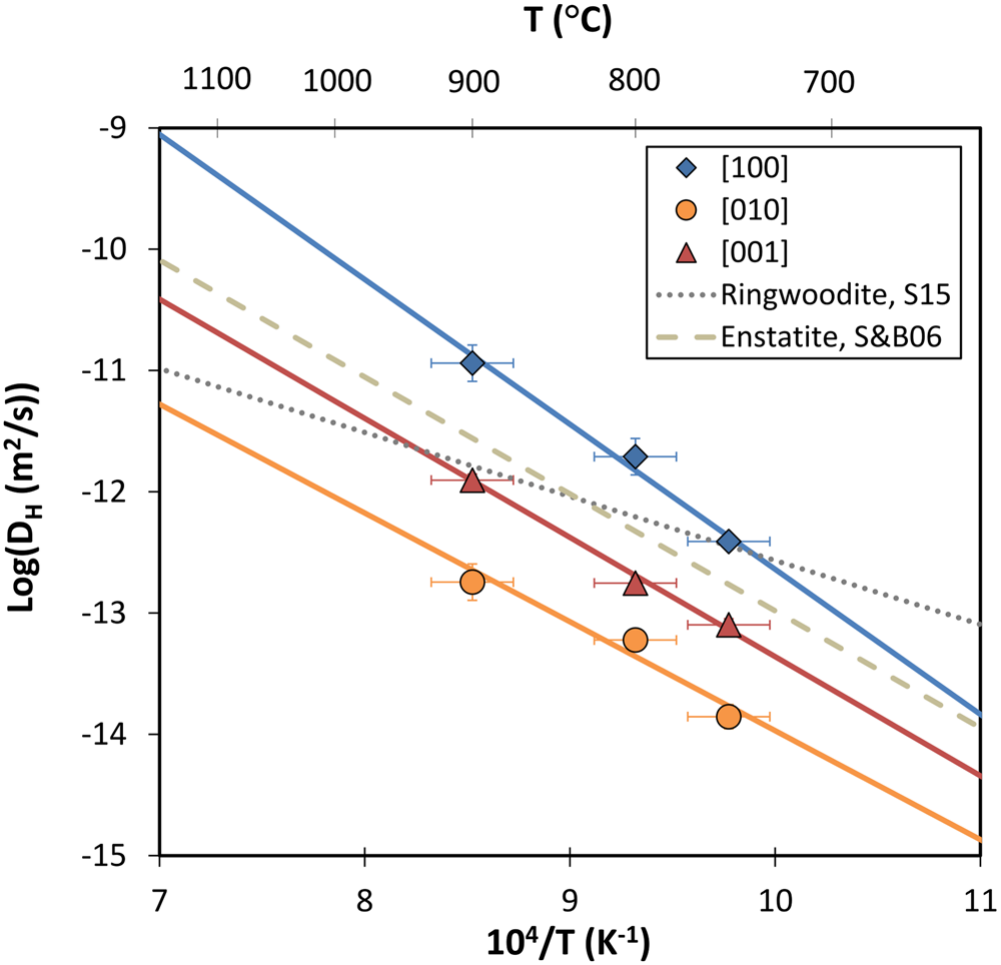
Arrhenius plot showing H self-diffusion coefficients as a function of inverse temperature. Errors for the diffusion coefficients are reported in Table 1 while a ±25 °C uncertainty of the experimental temperature is assumed. Solid lines represent weighted fittings of the experimental data along the three different orientations. Dash and dot lines show H self-diffusion coefficients for enstatite^28^ and ringwoodite^29^, respectively, from the literature. Temperatures in degrees °C are also shown at the top of the diagram.

The newly determined values are more precise and within the error of the previous self-diffusion coefficients for D_H,[100]_ determined by analyses using a CAMECA ims-6f SIMS on the same samples^30^ (Table 1). The temperature dependence of D_H,[010]_ and D_H,[001]_, determined for the first time for olivine in this study (Table 1, Fig. 3), are 1–2 log units lower than D_H,[100]_, consistent with hydrogen chemical diffusion coefficients reported for the redox exchange mechanism^11^. D_H,[100]_, D_H,[010]_ and D_H,[001]_ all increase exponentially with temperature between 750 to 900 °C, and were fit to the linear form of the Arrhenius equation:

2 $${{\rm{l}}{\rm{o}}{\rm{g}}(D}_{{\rm{H}},[\text{hkl}]})={{\rm{l}}{\rm{o}}{\rm{g}}(D}_{{\rm{H}},0})-{{\rm{H}}}_{{\rm{a}},[{\rm{h}}{\rm{k}}{\rm{l}}]}/({\rm{l}}{\rm{n}}(10)\ast {\rm{R}}{\rm{T}})$$

where D_H,0_ is the pre-exponential term (in m^2^/s), D_H,[hkl]_ is H self-diffusion coefficient along [hkl] direction (m^2^/s), H_a,[hkl]_ the activation enthalpy (in kJ/mol), T is the absolute temperature (K) and R is the gas constant (8.314 J/mol·K). The subscript in equation (2), e.g. [100], indicates the crystallographic orientation. Fits for the three principal orientations are shown in Fig. 3. The pre-exponential terms D_H,0_ = 10^−0.7±0.9^, 10^−5.0±0.9^ and 10^−3.5±0.4^ m^2^/s and activation enthalpies H_a,[hkl]_ = 229 ± 18, 172 ± 19 and 188 ± 8 kJ/mol were obtained for [100], [010], and [001] orientations, respectively. Diffusion profiles were resolved with higher spatial resolution, which allowed more precise determination of diffusion coefficients and consequently activation enthalpies. Thus, the activation enthalpy reported for D_H,[100]_ in this study is significantly higher and determined with higher precision than the previously reported value of 140 ± 30 kJ/mol^30^. The activation enthalpies for D_H_ in olivine determined here are comparable to enstatite^28^, suggesting a similar diffusion mechanism, but higher than ringwoodite^29^ (Fig. 3). All the experiments in this study were performed at a single pressure of 2 GPa, precluding determination of the activation volume. Activation volume is typically small for other ionic conduction in olivine^34, 35^, and a value of zero is subsequently used to compare against other laboratory results or geophysical observations at other pressures.

**Figure 4:**
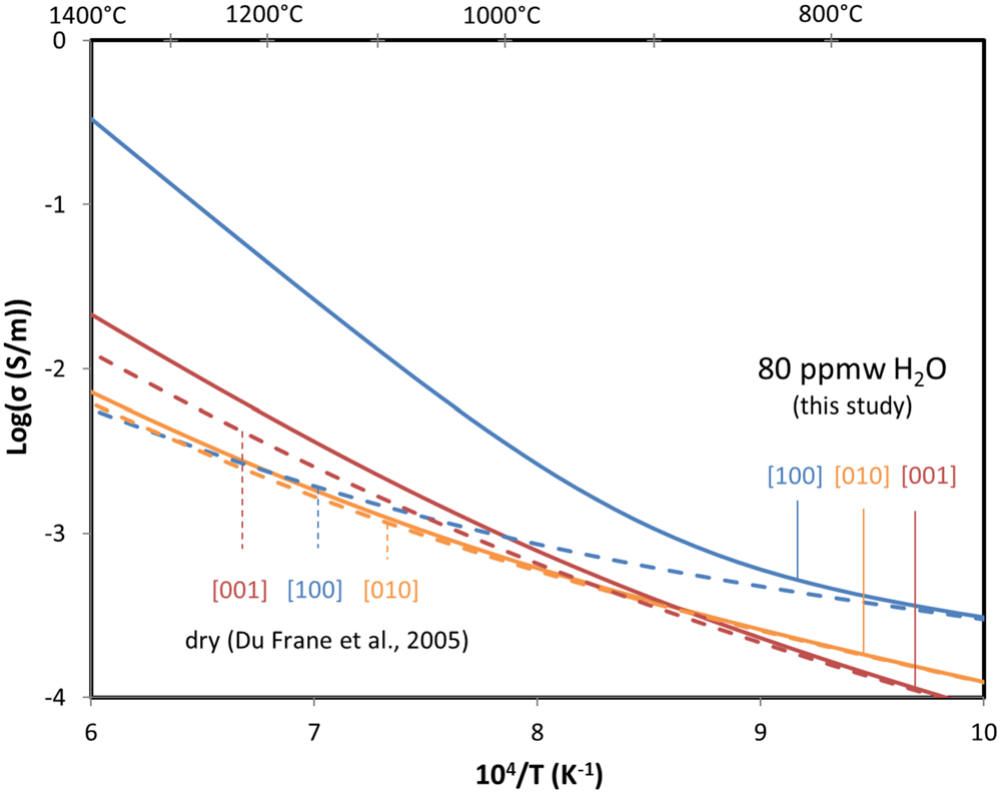
Total electrical conductivity of olivine as a function of inverse temperature (in Kelvin). Data for dry olivine^36^ along the three principal orientations are displayed by dashed lines while the calculated value for hydrous olivine (80 ppm wt H_2_O) are shown by blue ([100]), orange ([010]) and red ([001]) solid lines. Temperatures in degrees °C are also shown at the top of the diagram.

These new measurements of hydrogen self-diffusion in olivine as a function of temperature and orientation are used to calculate the contribution of H to olivine electrical conductivity by applying the Nernst-Einstein relation:

3 $${\sigma }_{{\rm{H}},[\text{hkl}]}={{\rm{f}}{\rm{D}}}_{{\rm{H}},[\text{hkl}]}{{\rm{C}}}_{{\rm{H}}}{{\rm{q}}}^{2}/(\text{kT})$$

where σ_H,[hkl]_ is the electrical conductivity of olivine due to the presence of H, f is a correlation function which is close to 1 (dimensionless), C_H_ is the concentration of H (1/m^3^), q is the charge (C), k is the Boltzmann constant (1.381 × 10^−23^ J/K) and T is temperature (K). The electrical conductivity of mantle olivine as a function of temperature (Fig. 4) is estimated by combining the conductivity of dry olivine with the influence of hydrogen, σ_H,[hkl]_:

4 $${{\rm{\sigma }}}_{\text{Total},[\text{hkl}]}={{\rm{\sigma }}}_{\text{Dry},[\text{hkl}]}+{{\rm{\sigma }}}_{{\rm{H}},[\text{hkl}]}$$

where σ_Total,[hkl]_ is the total conductivity of hydrated olivine and σ_Dry,[hkl]_ is the conductivity of dry olivine. Due to the relatively large activation enthalpies determined for hydrogen self-diffusion coefficients in comparison to that of other ionic conduction in olivine^34–36^, it is possible for hydrogen to become the dominant charge carrier at high temperatures when present in substantial concentrations (Fig. 4).

The electrical conductivity of dry olivine has limited anisotropy, <0.5 log units^36^. In contrast, electrical conductivity inferred for hydrous olivine is highly anisotropic (Fig. 4). For 80 ppm H_2_O, the olivine storage capacity calculated at ~120 km depth^37^, a maximum difference of ~0.3 log units is observed at low temperatures (up to ~900 °C) as a function of orientation (Fig. 4). At higher temperatures, however, the total anisotropy increases to approximately 2 log units (Fig. 4), due to the higher activation enthalpy for hydrogen self-diffusion and conduction, relative to other charge carriers in olivine. As a result, the contribution of H to electrical conductivity of hydrous olivine increases at higher temperatures (Fig. 3) with the highest enhancement observed for the [100] orientation (Fig. 4). Electrical anisotropy of up to 2 log units has been reported in the upper mantle in several magnetotelluric studies in different tectonic settings^38, 39^, and could potentially be attributed to hydrogen conduction in deformed mantle containing olivine that exibits preferred orientation.

The electrical conductivity of an isotropic, polycrystalline mantle composed of hydrous olivine can be approximated by a the geometric mean of the conductivities along the principle axes^40^:

5 $${{\rm{\sigma }}}_{{\rm{GM}}}={({{\rm{\sigma }}}_{[100]}\ast {{\rm{\sigma }}}_{[010]}\ast {{\rm{\sigma }}}_{[001]})}^{1/3}.$$

The cross-terms created by combining equations 4 and 5 are negligibly small, so the isotropic geometric mean for the contribution of hydrogen can simply be approximated as

6 $${\sigma }_{{\rm{H}}}={10}^{2.12}{\rm{S}}/{\rm{m}}\ast {{\rm{C}}}_{{\rm{H}}2{\rm{O}}}\ast {\exp }^{-187\text{kJ}/\text{mol}/({\rm{R}}{\rm{T}})}$$

where C_H2O_ is the concentration of H_2_O in ppm wt. In this case, Equation 6 can be combined as a sum with dry olivine data collected on polycrystalline samples as well^41^. This simple expression allows the contribution of hydrogen to be combined with any preferred model for the electrical conductivity of nominally anhydrous olivine.

**Figure 5:**
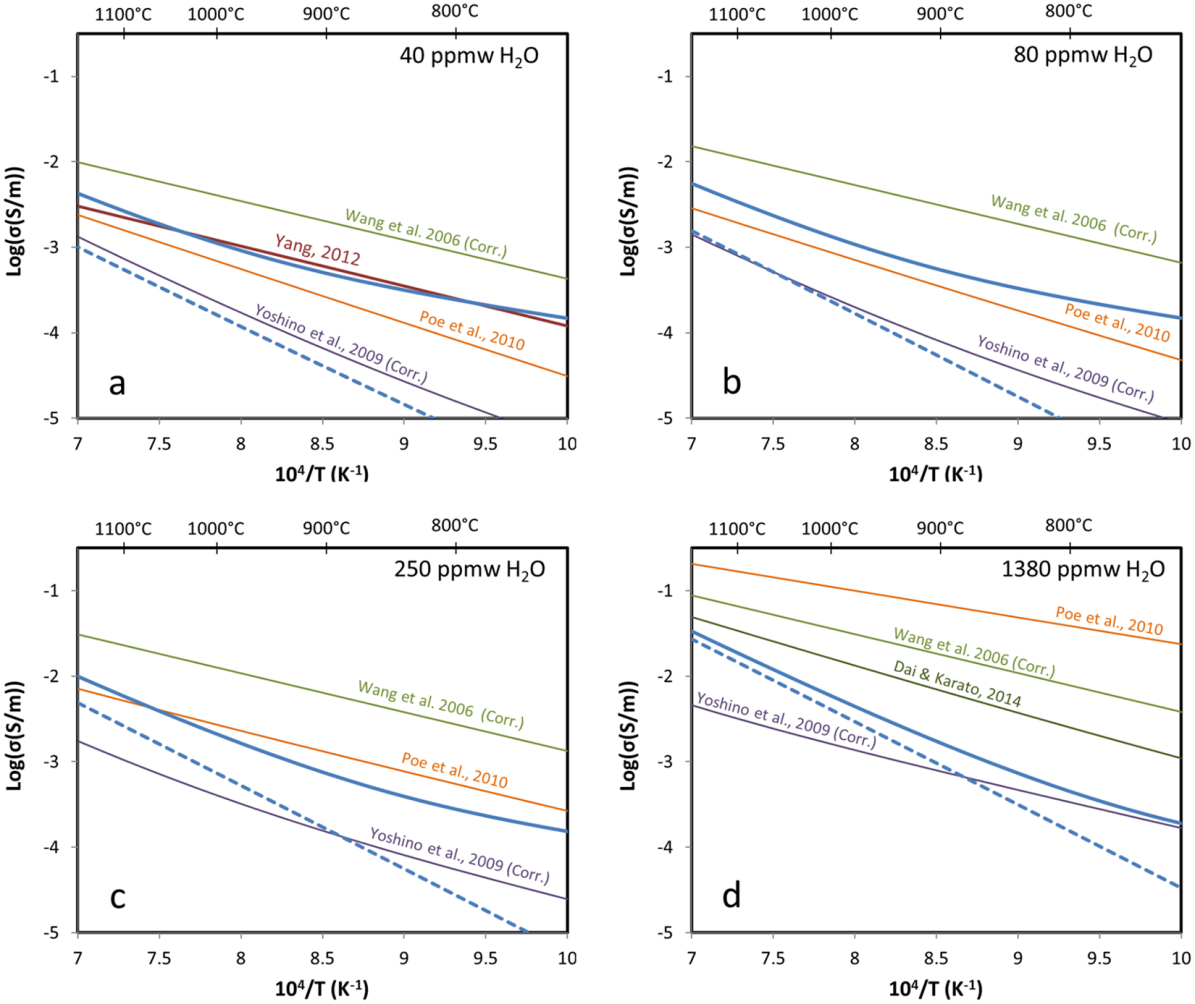
Electrical conductivity of hydrous olivine as a function of inverse temperature. Total electrical conductivity for hydrous olivine are calculated in this study using data for dry olivine (equations 4 and 5) from single crystal samples^36^ (blue solid line) or polycrystalline samples^41^ (blue dashed line). Total electrical conductivity from different studies in the literature^15, 23–26^ is also displayed. Panels (a), (b), (c) and (d) show values of 40, 80, 250 and 1380 ppm wt H_2_O. H_2_O contents from two studies^15, 23^ were corrected by a factor of 3 due to the use of different calibrations (see text). Temperatures in degrees °C are also shown at the top of the diagrams.

The electrical conductivity of olivine containing 40–1380 ppm wt H_2_O is compared with those resulting from direct determinations of electrical conductivity from previous studies with samples having a range of H_2_O concentrations (Fig. 5). H_2_O contents from two studies^15, 23^ were corrected by a factor of three due to the use of different calibrations that do not account for the effect of pleochroism in olivine on H_2_O quantification^42^. To be consistent with the other studies^24–26^, we correct to a previous olivine-specific calibration^43^, rather than the most recently reported one^44^.

## Discussion

The Nernst-Einstein theory has been used previously to simulate the influence of hydrogen on upper mantle electrical conductivity with models subsequently compared to magnetotelluric measurements of electrical conductivity anomalies in the asthenosphere^13, 30^. Utilizing H chemical diffusion coefficients for the [100], Karato^13^ concluded that the asthenospheric conductivity anomalies were consistent with olivine containing only limited amounts of H_2_O (<50 ppm wt). Du Frane and Tyburczy^30^ proposed a similar model, employing more appropriate H self-diffusion coefficients for the [100] direction, but only estimates for the [010] and [001], and concluded that H_2_O contents required to satisfy the observed conductivity anomalies in the asthenosphere likely exceeded the olivine storage capacity (up to 1 wt%). This recent effort^30^ is substantially improved in this study by the inclusion of experimentally determined H self-diffusion coefficients for D_H,[010]_ and D_H,[001]_, and higher precision coefficients for D_H,[100]_ (Table 1).

Use of the Nernst-Einstein relation and H diffusivity to estimate electrical conductivity in mantle olivine has been recently challenged^45, 46^. Based on high electrical conductivity results for hydrous olivine^15, 26^ – in comparison to other laboratories^14, 23–25^ – Karato^45^ argued that conductivity is largely dominated by a small subset of free protons resulting from an ionization reaction. Diffusion coefficients would therefore represent a harmonic average of all hydrogen species present, and consequently would be dominated by the diffusion of protons trapped in vacancy sites, and therefore limited by the diffusion of Fe and Mg ions that also occupy these sites. This argument implies that the vacancy and interstitial mechanisms for diffusion operate independently; however, this behavior is not expected to occur in naturally occurring olivine, where hydrogen predominantly occupies metal vacancies^11, 12, 27^, because interstitial ions will be frequently trapped by the chemical potential gradients of unoccupied vacancies^47^. Also, the experimental results used to support this argument^13^ imply diffusion coefficients for free protons that far exceed those of small polarons (i.e. electron holes)^30^, which is implausible based on the relative mass, ionic radius, and substantially higher site density of polarons in olivine.

To be consistent with chemical- and self-diffusion results for H in olivine published previously^11, 12, 30^ and presented in this study, it is likely that mixed vacancy-interstitial diffusion in olivine is controlled by a dissociative mechanism. In this case, an activated H dissociates from a metal vacancy and migrates as an interstitial particle until it is trapped again at another thermal vacancy^47^. This mechanism explains the relatively high hydrogen self-diffusion coefficients that are closer in magnitude to those of small polarons than metal vacancies within a crystal structure that predominantly contains sites that are metal vacancies. For the dissociative mechanism, the mean free terminal drift velocity of H would be the same in response to a chemical potential gradient or an electrical potential gradient (i.e. mobility), justifying the use of Nernst-Einstein relation for H diffusion and H diffusion data to model electrical conductivity.

The electrical conductivity values calculated for hydrous olivine in this study are in good agreement with most direct measurements^23–25^ for H_2_O contents at or below 250 ppm wt H_2_O, which is the storage capacity of olivine at ~240 km depth in the asthenosphere^37^ (Fig. 5a,b, and c). This model is in particularly excellent agreement with *in-situ* electrical conductivity measurements recently reported for olivine containing ~40 ppm wt H_2_O^25^ (Fig. 5a). There is good agreement with measurements reported for polycrystalline samples by Yoshino *et al*.^23^ when the model uses dry olivine values from polycrystalline samples^41^ rather than single crystal samples^36^. For their measurements on undoped samples that absorbed 50–120 ppm wt H_2_O from surrounding materials, the activation enthalpies fit in the temperature regime of ~1400–1700 °C (where hydrogen is expected to dominate) become significantly higher (2.14–2.25 eV or 206–217 kJ/mol)^23^ and consistent with values presented here for hydrogen self-diffusion (172–229 kJ/mol). For 1380 ppm wt, the model is in poor agreement with studies that included samples having very high H_2_O contents^15, 23, 24, 26^ that would only be possible in the lowermost portion of the upper mantle (Fig. 5d).

Electrical conductivity measurements on samples that have very high H_2_O contents may be problematic due to the potential influence of exsolved fluids on grain boundaries and interfaces within both samples and assemblies. Even the presence of small amounts of free fluids would mask the bulk electrical conductivity of olivine samples^20–22^. The contributions from such fluids may not produce noticeable hysteresis during temperature-cycling with deliberately high rates designed to minimize H_2_O loss from samples. Fluids would influence low temperature measurements where the conductivity of bulk olivine is expected to be low, and where the greatest discrepancies are observed amongst the published data-sets (Fig. 5). The presence of a fluid phase would lower the apparent activation enthalpies that are obtained in data fits, especially for higher H_2_O contents, which is an effect that has been required to fit data in some studies^22, 23^. This effect may be especially evident in samples with very high H_2_O contents (1380 ppm wt, Fig. 5d) that unexpectedly show little to no anisotropy and low activation enthalpies at low temperatures, and activation enthalpies that increase to become in better agreement with diffusion data in high temperatures regimes that are most relevant to the mantle^26^_._ These potential problems highlight the challenge in accurately determining temperature dependence and activation enthalpy of hydrous olivine electrical conductivity with *in-situ* high P-T experiments.

**Figure 6:**
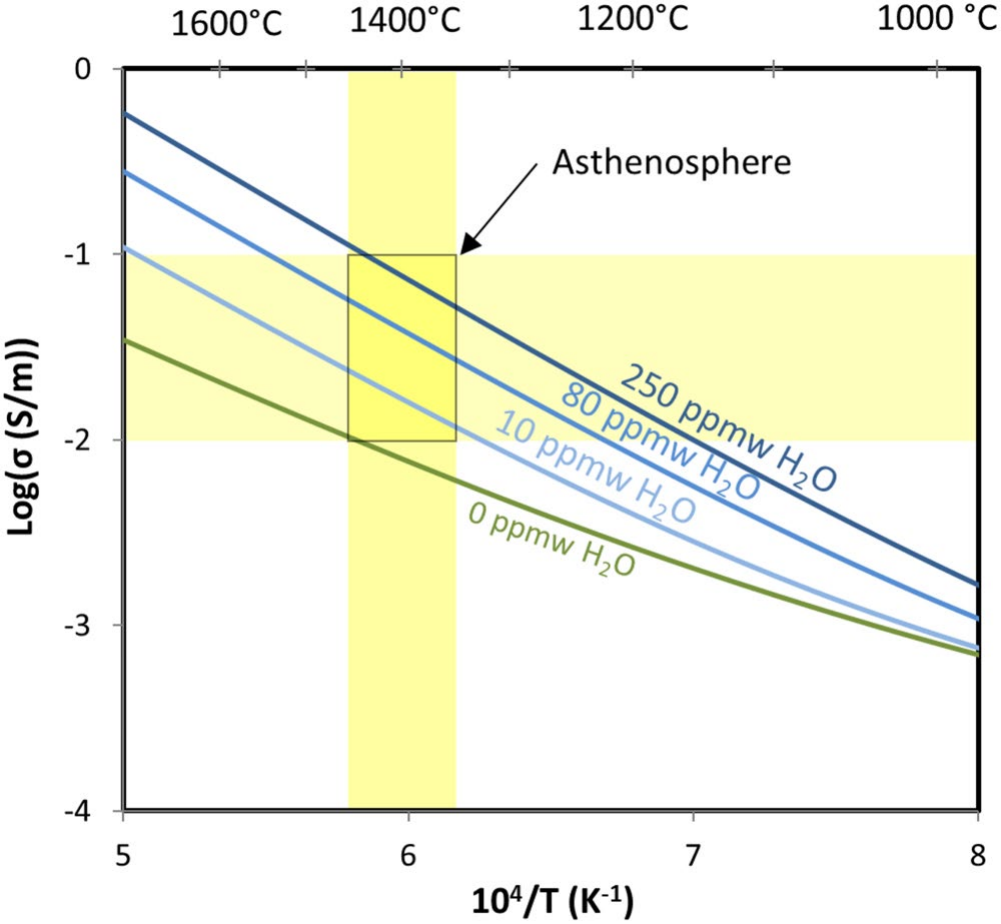
Olivine electrical conductivity as a function of inverse temperature (in Kelvin) and H_2_O content. Calculations are shown for dry olivine^36^ as well as hydrous (this study), with 10, 80 and 250 ppm wt H_2_O. Yellow bands display electrical conductivity anomalies measured in the asthenosphere^16–18, 48^ (horizontal) and adiabatic temperatures calculated at such depths (~1350–1450 °C^49^, vertical). Temperatures in degrees °C are also shown at the top of the diagram.

The H diffusion based model compares favorably with the range of electrical conductivities values observed in the asthenosphere by magnetotelluric studies (Fig. 6). High electrical conductivity anomalies observed beneath continental lithosphere, e.g. North East China^16^ and the French Alps^17^, are generally in the range of ~10^−2.0^ S/m. High electrical conductivity anomalies observed beneath oceanic lithosphere, e.g. Eastern Pacific Rise^18^ and the Cocos plate^48^, are generally in the range of ~10^−1.0^ S/m. For the depths of 120–240 km, roughly corresponding to where these anomalies are observed, pressure would be expected to increase from 4 to 8 GPa and temperature from 1350 to 1450 °C based on adiabatic calculations^49^. For these P-T conditions, olivine storage capacity would increase with depth from ~80 to 250 ppm wt^37^. The model in this study suggests that H_2_O contents up to 250 ppm wt can account for most of the electrical conductivity anomalies observed by magnetotelluric soundings^16–18, 48^ (Fig. 6).

Utilizing H partitioning parameterizations among upper mantle mineral phases and predicted mineral abundances^50^, the H_2_O content expected in olivine can be calculated for mantle assemblage with different overall H_2_O contents. The highest end of the range of MORB and OIB mantle sources reported are approximately 200 and 1000 ppm wt respectively, ﻿e.g. ﻿Saal *et a﻿l.﻿* and Bureau *et al.* 51, 52. For a mantle assemblage containing either 200 or 1000 ppm wt H_2_O at depths between ~120–240 km, the olivine is predicted to contain approximately 90–180 and 470–900 ppm wt H_2_O, respectively. The upper limit of H_2_O contents inferred for olivine beneath oceanic lithosphere by this model therefore predicts mantle H_2_O contents that are close to those expected in MORB mantle sources, and well below OIB mantle sources (Fig. 6). This demonstrates that experiments measuring H self-diffusion coefficients are a promising tool to investigate the effect of H on electrical conductivity of mantle materials, and interpret magnetotelluric measurements to probe the presence of H in the Earth’s interior.

## Methods

### Samples

Oriented single crystals of San Carlos olivine were pre-annealed via the redox exchange mechanism^11^ in high P-T piston cylinder experiments at 2 GPa and between 750 to 900 °C^30^. Heating/cooling rates to the target anneal temperature were sufficiently high such that the effective additional annealing times are calculated to be negligible (estimates provided in Table S1)^53^. Samples were hydrated in isotopically normal water at run conditions, quenched and then annealed at the same run conditions in the presence of deuterated water allowing deuterium (^2^H) to diffusively exchange with hydrogen (^1^H). The method imposes a hydrogen isotopic gradient in the absence of a hydrogen concentration gradient. ^2^H is predicted to diffuse similarly to ^1^H but with some diffusive fractionation (with D(^1^H) = D(^2^H)/√2). Samples were all cut from one single, large crystal that was oriented by Laue back-scattered x-ray diffraction. From this procedure, olivine cuboids whose edges of ~1 mm length were aligned along the three principal orientations [100], [010] and [001]. Further details regarding the samples and experimental setup are reported previously^30^.

## NanoSIMS

Hydrogen self-diffusion along all three principal orientations in olivine were measured as a gradient in ^16^O^2^H^-^ normalized to ^28^Si^-^ using a CAMECA NanoSIMS 50 housed at Lawrence Livermore National Laboratory (LLNL). The unique features of the NanoSIMS allow for both the high spatial resolution and detection sensitivity that are required to resolve the extremely short diffusion profiles of trace elements, such as H, along [010] and [001] directions.

The olivine crystals used in these experiments were polished and pressed into indium mounted in holes inside an aluminum disc. The entire mount was sputter coated with ~5 nm of gold. The analyses were performed using a primary Cs^+^ beam of ~10 pA that was focused on the sample surface resulting in the ejection of secondary ions. A normal incidence electron flood gun (~100 nA e^−^ in ~60 µm diameter area) was used for charge compensation during the analyses. For each analysis, the surface was sputtered prior to data collection for 3 minutes over a 4 × 4 µm^2^ square, which was followed by ~7 minutes of collecting time from a 2 × 2 µm^2^ area at the center of the previously sputtered region (Fig. 1). This approach reduced the contribution of surface H_2_O to the analyses to background levels (≤5 × 10^−6 16^O^2^H^−^ /^28^Si^−^). ^16^O^1^H^−^, ^16^O^2^H^−^, ^28^Si^−^ and ^12^C_2_^−^ were simultaneously collected on electron multipliers in pulse counting mode. Despite these efforts, ^16^O^2^H^−^ profiles are used to fit diffusion coefficients, because ^16^O^1^H^−^ measurements are more challenging and generally have higher errors associated with tuning and alignment of the ion beam, and interference from background levels and surface contamination of ^1^H. The ^12^C_2_^−^ count rate was used to identify analysis locations that included cracks or impurities (Fig. 1), for which the data were not used. The analyses were conducted while maintaining vacuum conditions <3 × 10^−10^ Torr or lower in the sample chamber.

The ^2^H (D) analytical profiles, quantified as ^16^O^2^H^−^ /^28^Si^−^, begin at the sample edge and progress toward the center of the crystal, on a path perpendicular to the edge. To avoid interference with diffusion from other orientations, profiles were positioned near the center of each edge (Fig. 1). In all profiles, the ^2^H content decreased from the edge of the crystal toward the center. ^2^H diffusion profiles along the [100] orientation are much longer than those for [010] and [001] orientations (Fig. 2 and Supplementary Figures S1 and S2). Consistent with diffusive isotopic exchange of ^2^H-^1^H, the ^1^H diffusion profiles along [100] are complementary to ^2^H, with concentrations decreasing from the center of the crystal toward the edge^30^ (Supplementary Figure S4). This observation implies that the ^2^H diffusion profiles can be used to approximate ^1^H self-diffusion.

The dataset presented here is limited to three experiments at three different temperatures, but further work will be needed to increase the robustness of knowledge of hydrogen self-diffusion in olivine. Additional experiments over a wider spread and repeat temperatures would reduce uncertainties in activation enthalpies, and therefore extrapolations to higher temperatures. It is possible for there to be slight crystal to crystal variations in hydrogen diffusion coefficients measured in NAMs^54^, thus studies over a broad distribution crystals are needed to determine the extent that this is true for olivine. Finally, experiments with zero-duration dwells at the target would confirm there are no other unforeseen contributions to the diffusion profiles that are observed (e.g. during heating/cooling).

### Scanning Electron Microscopy and Electron Microprobe Analyses

SEM images were collected to investigate the array of analyses forming the different profiles. This procedure is essential to determine the exact distance from the edge of the crystal of the craters formed by the NanoSIMS analyses, and to identify the presence of sources of impurities such as cracks or fractures in the analyzed pits that could invalidate a measurement. The images were collected with an FEI INSPECT F SEM at LLNL, which was operated with an accelerating voltage of 15 kV. Both secondary electron and backscatter electron images were collected to evaluate topographical and compositional details of the analyzed areas (Fig. 1).

Electron microprobe (EMP) wavelength dispersive x-ray analyses were also performed to accurately determine the composition of olivine and exclude from analyses any areas of chemical zonation in the investigated samples. No significant crystal growth was observed at the boundaries of samples under electron microprobe. The analyses were conducted by means of a JEOL JXA 8200 electron microprobe at LLNL, which is equipped with 5 separate spectrometers. Spot analyses (~1 µm diameter) were conducted on the crystals at 15 kV accelerating voltage and 15 nA beam current. Counting time was 20 s on peak position and 10 s on each side for background, and quantitative analyses were made possible by means of MgO, Fe_2_O_3_ and diopside standards. The data were corrected following the CITZAF procedure^55^. Chemical composition of the crystal used in experiment PC33 is reported in Table S2.

## Electronic supplementary material

Supplementary information
