# Supplementary material for: Hydrogen self-diffusion in single crystal olivine and electrical conductivity of the Earth’s mantle
Source: Sci Rep. 2017 Jul 13;7:5344. doi: 10.1038/s41598-017-05113-6 (PMC5509734; doi:10.1038/s41598-017-05113-6)
Supplement: Supplementary file 1 — Supplementary information [file 41598_2017_5113_MOESM1_ESM.pdf]

Supplementary information

Hydrogen self-diffusion in single crystal olivine and electrical conductivity of the Earth's mantle

Davide Novella[1,*,+], Benjamin Jacobsen[1], Peter K. Weber[1], James A. Tyburczy[2], Frederick J. Ryerson[1] and Wyatt L. Du Frane[1,*]

Supplementary Table S1

Table S1: Estimates of effective additional anneal times (Δt) due to heating/cooling.

[hkl]	anneal T (°C)	ΔH (kJ/mol)	anneal t (m)	Δt(m)*
[100]	900	229	10.0	0.51
[100]	800	229	15.0	0.43
[100]	750	229	60.0	0.39
[010]	900	172	10.0	0.68
[010]	800	172	15.0	0.57
[010]	750	172	60.0	0.51
[001]	900	188	10.0	0.62
[001]	800	188	15.0	0.52
[001]	750	188	60.0	0.47

*Approximated as $\Delta t \approx (1/\alpha_{up} + 1/\alpha_{down})RT^2/H_{a,[hkl]}$ where α_{up} is the heating rate ~1.67 K/s, α_{down} is the quench rate ~100 K/s, R is the gas constant, $H_{a,[hkl]}$ is activation enthalpy[53].

Supplementary Table S2

Table S2: chemical composition of olivine

Al_2O_3	0.01 (0.01)
MnO	0.15(0.01)
MgO	47.52 (0.38)
FeO	11.25 (0.04)
SiO_2	40.94 (0.44)
NiO	0.35 (0.01)
CaO	0.10 (0.01)
Total	100.32 (0.65)
Fo #	0.88

Averages from 14 analyses. 1σ given between brackets.

Supplementary Figure S1

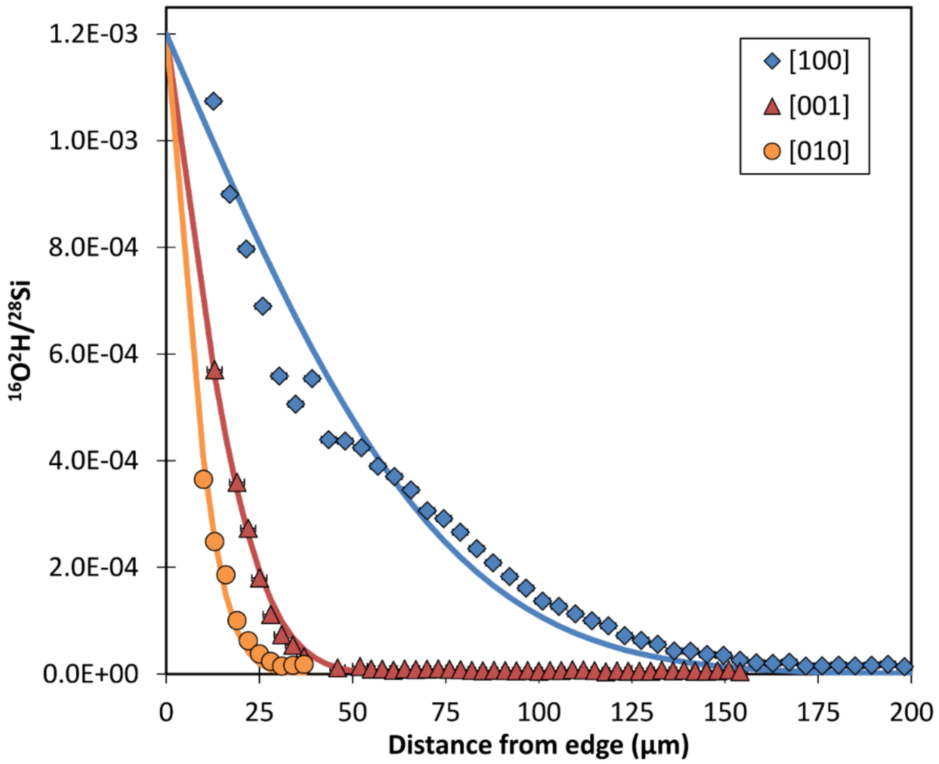

Fig. S1: Deuterium diffusion profiles along [100] (blue diamonds), [001] (red triangles) and [010] (yellow circles), with data-fits to Equation 1 shown as lines in corresponding colors for experiment PC28 (2 GPa, 800 ˚C).

Supplementary Figure S2

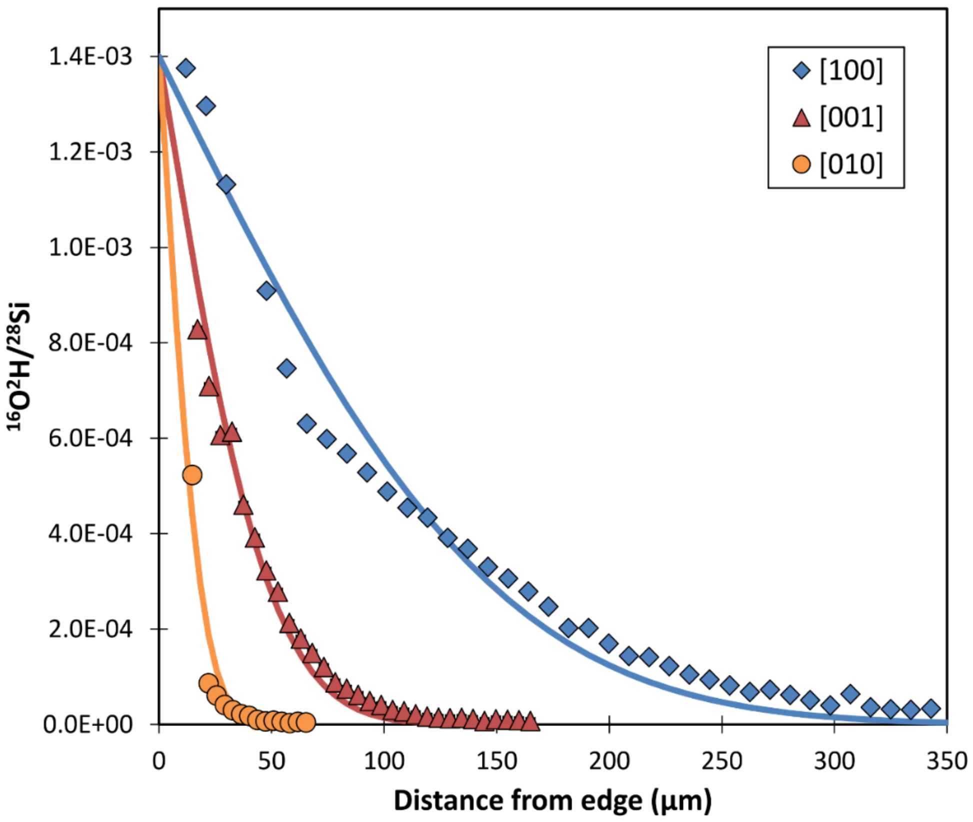

Fig. S2: Deuterium diffusion profiles along [100] (blue diamonds), [001] (red triangles) and [010] (yellow circles), with data-fits to Equation 1 shown as lines in corresponding colors for experiment PC33 (2 GPa, 900 ˚C).

Supplementary Figure S3

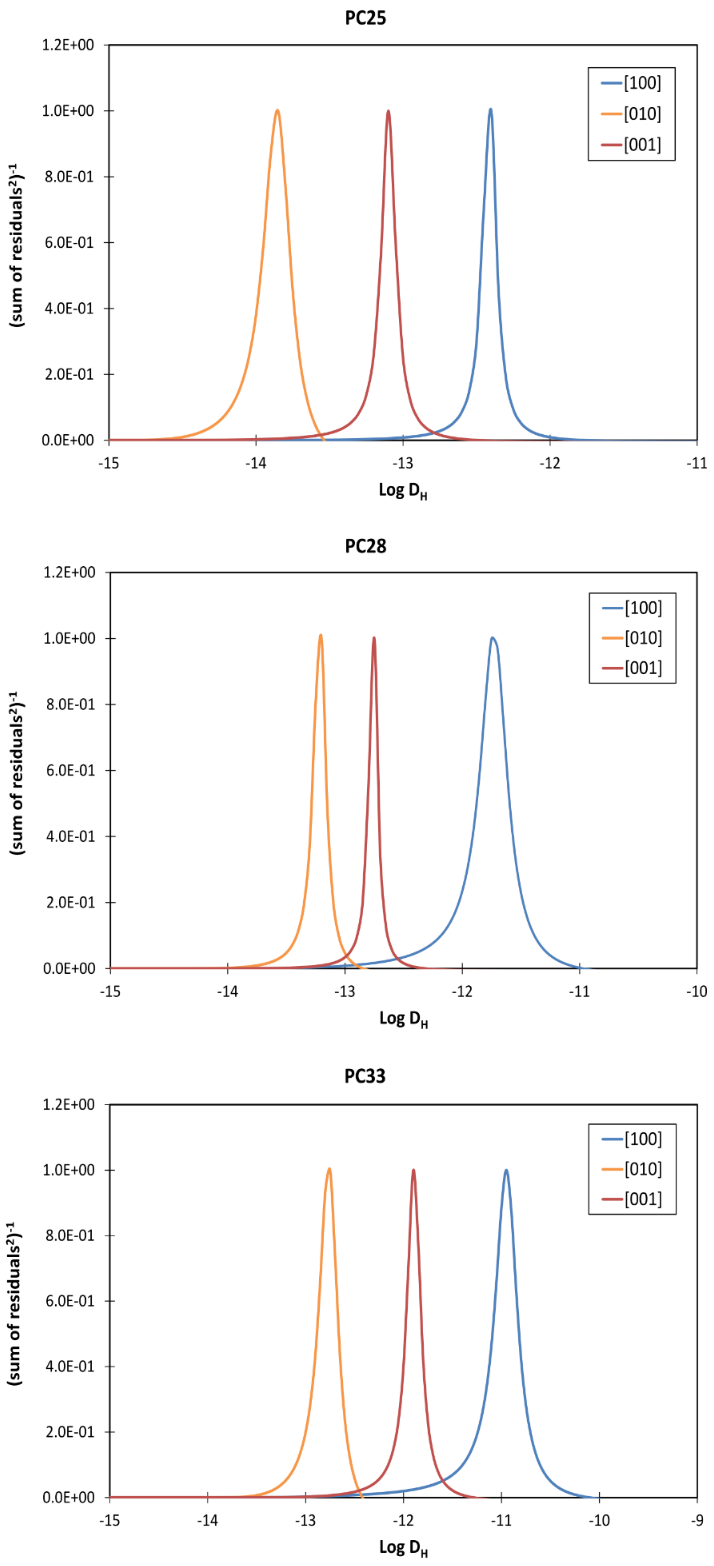

Figure S3: Sum of residual square (normalized to 1) from fittings along [100], [010] and [001] for the 3 experiments. The fitting errors were calculated as 1σ from each solution.

Supplementary Figure S4

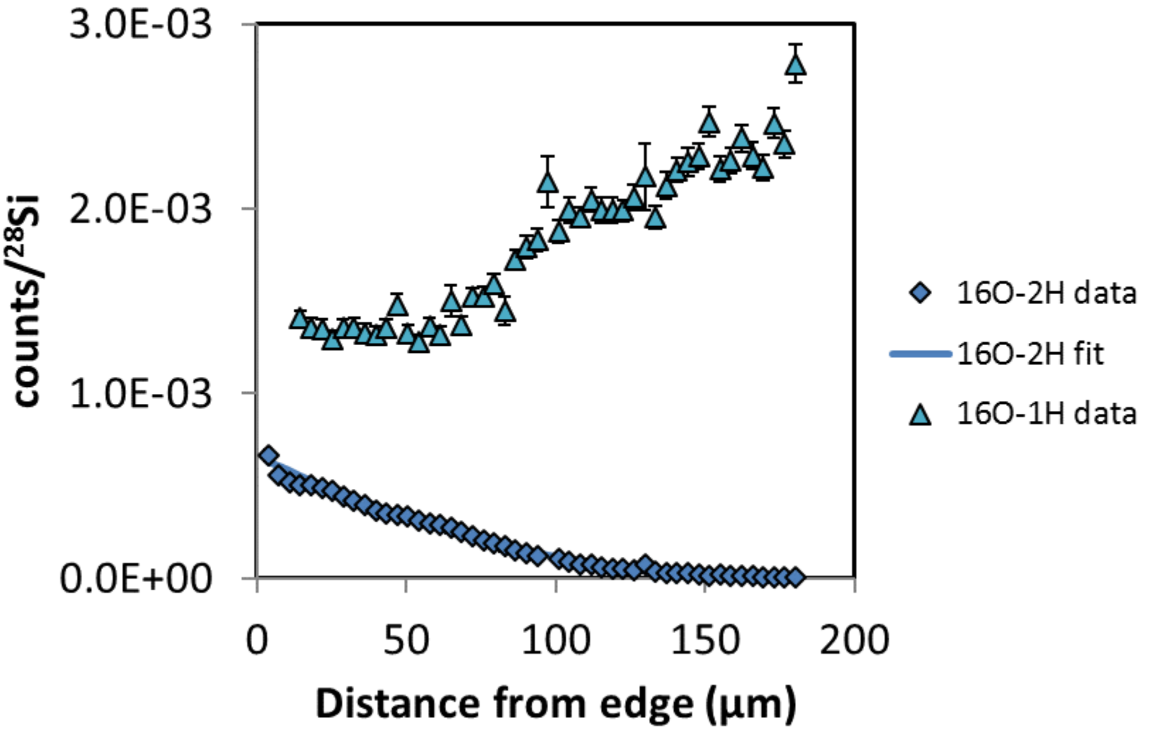

Figure S4: NanoSIMS counts of $^{16}O^{2}H$ (dark blue diamonds) and $^{16}O^{1}H$ (light blue triangles) normalized to ^{28}Si along [100] for PC25 sample after ^{2}H-^{1}H exchange at 2 GPa, 750 °C, for 60 min. Due to matrix effects common for SIMS analyses, the relative counts of $^{16}O^{2}H$ and $^{16}O^{1}H$ are not directly comparable to ^{2}H and ^{1}H concentrations. Determining absolute concentrations of ^{2}H and ^{1}H requires comparison against olivine standards, which were not available for this study. The $^{16}O^{1}H$ data has relatively larger scatter in comparison to that of $^{16}O^{2}H$ due to background and surface contamination in pits, scratches, or the edge between the sample and surround indium. However, the data does demonstrate that ^{1}H leaves the sample, and ^{2}H enters the sample, which is consistent with ^{2}H-^{1}H exchange.
